# The effects of continuous prostacyclin infusion on regional blood flow and cerebral vasospasm following subarachnoid haemorrhage: study protocol for a randomised controlled trial

**DOI:** 10.1186/1745-6215-13-102

**Published:** 2012-07-02

**Authors:** Rune Rasmussen, Jørn Wetterslev, Trine Stavngaard, Jane Skjøth-Rasmussen, Per Olof Grände, Niels Vidiendal Olsen, Bertil Romner

**Affiliations:** 1 Department of Neurosurgery, Copenhagen University Hospital, 9 Blegdamsvej, Copenhagen, Denmark; 2 Copenhagen Trial Unit, Centre for Clinical Intervention Research, Copenhagen University Hospital, Rigshospitalet 9 Blegdamsvej, Copenhagen, Denmark; 3 Department of Radiology, Copenhagen University Hospital, 9 Blegdamsvej, Copenhagen, Denmark; 4 Department of Anaesthesia and Intensive Care, Lund University Hospital, 4 Getingevägen, Lund, Sweden; 5 Department of Neuroanesthesiology, Copenhagen University Hospital, Blegdamsvej 9, Copenhagen, Denmark

**Keywords:** Subarachnoid haemorrhage, Prostacyclin, Epoprostenol, Vasospasm, Delayed ischaemic neurological deficit

## Abstract

**Background:**

One of the main causes of mortality and morbidity following subarachnoid haemorrhage (SAH) is the development of cerebral vasospasm, a frequent complication arising in the weeks after the initial bleeding. Despite extensive research, to date no effective treatment of vasospasm exists. Prostacyclin is a potent vasodilator and inhibitor of platelet aggregation. In vitro models have shown a relaxing effect of prostacyclin after induced contraction in cerebral arteries, and a recent pilot trial showed a positive effect on cerebral vasospasm in a clinical setting. No randomised, clinical trials have been conducted, investigating the possible pharmacodynamic effects of prostacyclin on the human brain following SAH.

**Methods:**

This trial is a single-centre, randomised, placebo-controlled, parallel group, blinded, clinical, pilot trial. A total of 90 patients with SAH will be randomised to one of three intervention arms: epoprostenol 1 ng/kg/min, epoprostenol 2 ng/kg/min or placebo in addition to standard treatment. Trial medication will start day 5 after SAH and continue to day 10. The primary outcome measure is changes in regional cerebral blood flow from baseline in the arterial territories of the anterior cerebral artery, medial cerebral artery and the posterior cerebral artery, measured by CT perfusion scan. The secondary outcomes will be vasospasm measured by CT angiography, ischaemic parameters measured by brain microdialysis, flow velocities in the medial cerebral artery, clinical parameters and outcome (Glasgow Outcome Scale) at 3 months.

**Trial registration:**

Clinicaltrials.gov NCT01447095.

## Background

Subarachnoid haemorrhage (SAH) accounts for only 5% of strokes, but due to the poor prognosis and lower patient age than for patients with ischaemic stroke, the loss of productive life years due to SAH approaches that for ischaemic stroke and intracerebral haemorrhage [1].

One of the main causes of mortality and morbidity following SAH is the development of delayed ischaemic neurological deficit (DIND) [2]. DIND occurs in approximately 30% of all patients and often develops within the first 2 weeks after the haemorrhage, with maximum onset between days 4 and 10 [3,4]. The presumed cause of DIND is the development of cerebral vasospasm (CV), which can be demonstrated by angiography in 70% of patients following SAH [4]. However, not all patients with angiographic vasospasm suffer DIND and DIND can occur without evidence of arterial narrowing [3]. Several mechanisms have been suggested as additional causes of DIND, e.g. microthrombosis [5]; however angiographic vasospasm is still regarded as the most important factor.

The pathophysiology behind the development of CV is complex and remains poorly understood, but factors related to the vascular endothelium and the smooth muscle cell play a crucial role [6]. Several intervention options, including HHH (hypertension, hypervolemia, hemodilution), calcium antagonists, angioplasty, endothelin-receptor antagonists and statins have been used to prevent or treat vasospasm, but to date treatments with convincing effects are lacking.

Prostacyclin (PGI_2_) is an endogenous substance released from the vascular endothelium. It is a potent vasodilator and inhibitor of leukocyte activation, platelet aggregation and leukocyte-endothelial interactions [7]. A reduced amount of prostacyclin has been found in cerebral vessels in monkeys following SAH, and an imbalance in the prostacyclin-prostaglandin ratio has been proposed as a cause of vasospasm [8,9].

In vitro studies have demonstrated a relaxing effect of low-dose prostacyclin on cerebral vessels after induced contraction [9-11], and animal studies have demonstrated a positive effect of PGI2 on cerebral blood flow in rats with traumatic brain injury [12,13].

A limited number of studies exist investigating the possible effects of prostacyclin on cerebral vessels in a clinical setting. A case report has described significant recovery from segmental vasoconstriction in the brain after administration of prostacyclin 0.9 ng/kg/min [14]. In a study of five patients with traumatic brain injury, normalization of ischaemic parameters measured by cerebral microdialysis was seen after administration of low-dose prostacyclin (0.5-1 ng/kg/min) [15]. However, a randomized, blinded trial of 48 patients with traumatic brain injury was not able to reproduce this effect [16].

A recent pilot trial from 2009 investigated the effect of low-dose prostacyclin on vasospasm following SAH. In five patients with documented cerebral vasospasm, prostacyclin 0.5 ng/kg/min was administered and flow velocities in the medial cerebral artery were subsequently monitored by transcranial Doppler. In all patients, flow velocities decreased markedly as an expression of the resolution of vasospasm [17].

To date, no randomised, clinical trials have been conducted investigating the possible pharmacodynamic effects of prostacyclin on the vascular bed of the human brain following SAH (search details in Table 1).

**Table 1:** Search details

	**Search string 1**	**Search string 2**	**Combined**
PubMed	Subarachnoid haemorrhage (MeSH term)	Epoprostenol (MeSH term)	
	14664	11612	21
Embase	Subarachnoid haemorrhage	Prostacyclin	
	25363	26454	88
Cochrane central	Subarachnoid haemorrhage	Prostacyclin	
	15	8	0

Number of search hits indicated below each term. Automatic explode function on.

Although the use of prostacyclin in the treatment of cerebrovascular disease so far has only been experimental, synthetic prostacyclin (epoprostenol) has been used as a standard treatment for pulmonary hypertension for years, in doses 2–20 times higher than doses used in this trial. The side effects of these high doses are well known and include the risk of hypotension due to vasodilation and the risk of bleeding. The experience using low-dose prostacyclin in the treatment of neurovascular disease is limited. In the above-mentioned trial of 48 patients with traumatic brain injury, no episodes of bleeding or hypotension were observed.

## Methods

### Overview

This trial is a single-centre, randomised, placebo-controlled, parallel group, blinded, clinical trial. The trial is an explorative, pilot trial designed to investigate the feasibility and possible effects of low-dose prostacyclin on the primary outcome of regional blood flow and vasospasm in the human brain following SAH.

The trial is being conducted at Rigshospitalet, Copenhagen University Hospital, Neurointensive Care Unit, Denmark. The trial was approved by the Danish ethical committee on human research (reference no. H-1-2011-087), the Danish Medicines Agency (EudraCT 2011-002798-5) and registered on http://www.clinical.trials.gov (reference no. NCT01447095), and will be carried out in compliance with the Declaration of Helsinki. The trial will be reported in compliance with the CONSORT statement ( http://www.consort-statement.org).

**Figure 1:**
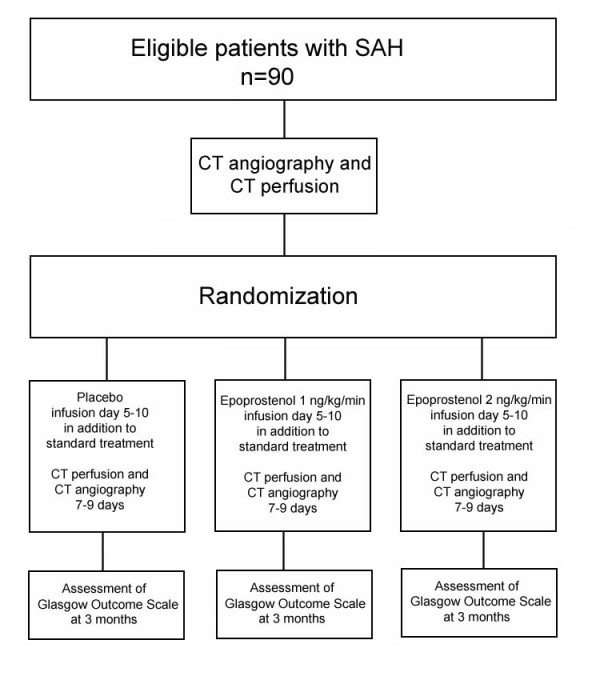
Flow diagram.

A total of 90 patients will be randomized to one of three intervention arms, flowchart in Figure 1. No interim analysis will take place. In- and exclusion criteria are listed in Table 2.

**Table 2:** In- and exclusion criteria


Inclusion criteria	SAH verified by CT
	Fisher grade 3 + 4
	Aneurysm treated by surgical clipping or endovascular coiling
	Age > 18 years
	WFNS grade 1-4
Exclusion criteria	Pregnancy/lactation
	Renal failure
	Heart failure
	Bleeding diathesis
	Liver failure
	Major complication during endovascular procedure or surgery
	Previous SAH
	SAH on basis of PICA aneurysm

### Patient enrolment and randomization

Patients with SAH admitted at our institution with a significant amount of blood in the subarachnoid space (Fisher grade 3 + 4) are potential candidates for the trial. After informed consent, patients are randomized to either a continuous infusion of epoprostenol 1 ng/kg/min, or epoprostenol 2 ng/kg/min, or placebo (drug solvent). Concealed allocation is achieved by randomisation and allocation by specially assigned nurses without patient contact. Randomisation is done using a computer-generated allocation list from http://www.randomization.com, only accessible by the specially assigned nurses not involved in patient care in any circumstances. When a patient is included, the nurse is contacted by telephone and the patient is subsequently randomised. The nurse will then prepare the blinded trial medication according to the allocated intervention.

### Epoprostenol intervention

Trial medication will start on day 5 after SAH and continue to day 10, as risk of vasospasm is at its highest in this time interval. Trial medication is given in addition to standard treatment and is administered as a continuous intravenous infusion. Epoprostenol 500 mg (Flolan R, GlaxoSmithKline) will be obtained and diluted only with original solvent. Solutions of 5,000 ng/ml, 10,000 ng/ml and 0 ng/ml (pure solvent) will be prepared. All solutions are colourless. Trial medications are prepared in syringes, ready for infusion. The syringes are marked with the patient ID, the same infusion rate for all three solutions and date, and are then transported to the neuro-intensive care unit. The infusion rate is determined by patient weight, according to the treatment schedule. Only specially assigned nurses will handle the trial medications until the transport to the neuro-intensive care unit. Thereafter, the trial medication is administered by the nursing staff on duty. Syringes will be kept refrigerated and will be changed every 8 h. If serious adverse events (hypotension, bleeding episodes or anaphylaxis) occur, the trial medication will be stopped.

### CT perfusion

Baseline CT perfusion (CTP) will be performed at day 3 (± 1 day) after SAH. CTP during the intervention is performed at day 8 (± 1 day) after SAH. All scans are performed on a Phillips Brilliance scanner.

The CTP scan covers a 4-cm area selected at the level of the basal ganglia. CTP data acquisition consists of a 60-s series during intravenous administration of iodinated contrast material (details in Table 3). All images are stored on a database to be subjected to later blinded analyses of the primary outcome measure.

**Table 3:** Scanning protocol for CT perfusion (CTP) and CT angiography (CTA)

	**CTP**	**CTA**
Amount of contrast	40 ml	70 ml
Injection rate	4 ml/s	4 ml/s
Acquisition parameters	80 kVp and 110 mAs	120 kVp/190 mA
Section thickness	5 mm	0.67 mm

Three regions of interests (ROIs) will be placed in each hemisphere: The cortex of the arterial territory of the anterior cerebral artery, the middle cerebral artery and posterior cerebral artery. Regional cerebral blood flow (rCBF) for each ROI is calculated using the deconvolution method (Philips Medical Systems, EBW workstation v. 3.5).

### CT angiography

Baseline CT angiography (CTA) will be performed routinely at admission. CTA during intervention is performed day 8 ± 1 day after SAH, 10 min after CTP. A caudocranial scanning direction is selected, covering the entire brain down to 1 cm below the foramen magnum (details in Table 3).

CTA raw data are reformatted in axial, sagittal and coronal maximal intensity projection (MIP) images. MIP images are reviewed by an experienced neuroradiologist for vasospasm and qualified as absent, mild/moderate or severe.

### Brain microdialysis

Microdialysis catheters (CMA 70 10-mm membrane length, 20-kd cutoff) are inserted into the white matter in the arterial territory of the aneurysm-bearing artery.

The microdialysis catheters are perfused (Perfusion Fluid, CMA Microdialysis) at a rate of 0.3 l/min, and the perfusates are collected in capped microvials at 2-h intervals. The samples are analysed for glucose, pyruvate, lactate and glycerol (ISCUS Microdialysis Analyzer). The area under the curve is calculated for each substance before, during and after intervention.

### Follow-up

Three months after SAH, all patients will be contacted by phone or are seen in our outpatient clinic. Clinical outcome will be recorded using the Glasgow Outcome Scale (GOS).

### Outcome measures

Two types of regions of interest (ROIs) are considered equally biological relevant and accordingly the two co-primary outcomes will focus on:

1) regions with decreased blood flow (<30 ml/100 g/min) at baseline

2) regions with decreased blood flow during the intervention (measured on day 8 ± 1 day).

Regions with normal regional cerebral blood flow (rCBF) both at baseline and after the intervention period will be considered to have normal regional blood flow during and after the intervention period.

Thus, two separate analyses will be performed:

1. ROIs with decreased flow values at the baseline scan: The mean rCBFs for these ROIs are calculated for each patient. Thereafter, the mean values of the corresponding ROIs on the intervention scan are calculated. The averages of differences of these mean values are compared between intervention groups using analysis of covariance.

2. ROIs with decreased flow values at the intervention scan: The mean rCBFs for these ROIs are calculated for each patient. Thereafter, the mean values of the corresponding ROIs at the baseline scan are calculated. The averages of differences of these mean values are then compared between intervention groups using analysis of covariance.

Secondary outcome measures will be:

Radiographic vasospasm measured by CT angiography

Microdialysis parameters (as described above)

Occurrence of DIND as defined by Vergouwen et al. [3]. Any occurrence of DIND will be recorded by the physician on duty and verified by the investigator on a daily basis.

Mortality and morbidity (GOS) at 3 months

Flow velocities in a. cerebri media measured with transcranial Doppler

Serum levels of S100b (brain damage biomarker) in peripheral blood

### Adverse reactions

Serious adverse reactions (SARs) and suspected unexpected serious adverse reactions (SUSARs) will be registered according to the protocol approved by the Danish Medicines Agency (DMA). As all patients with SAH are expected to experience events due to their critical illness; only SARs and SUSARs will be reported to health authorities in compliance with the national legislation.

### Monitoring

This trial is monitored by The GCP unit at Copenhagen University Hospital. As the intervention is a low-dose epoprostenol infusion with low risk and the trial inclusion period as well as the sample size are limited, no data safety and monitoring committee has been established in accordance with the protocol and the DMA.

### Sample size estimation

Difference in changes of regional cerebral blood flow day 8 (± 1 day) from baseline will be the two co-primary outcome measures of the trial. Based on a type 1 error risk of 2.5%, a type 2 error risk of 20% (a power of 80%), a standard deviation of 25% and the possibility to detect or reject a 20% difference in the change of average regional CBF from baseline between the intervention groups, 30 patients per intervention arm have to be randomised.

### Statistical analyses

Analysis of covariance will be performed for the primary outcome measure to allow for individual levels at baseline of the covariates age, previous neurological status and the mean rCBF for ROIs affected at baseline to be taken into account [18]. Ordinal logistic regression will be performed for the effect on GOS pending the intervention group using the proportional odds approach; the model considers every possible way in which an ordinal scale can be dichotomized, assuming that the odds ratio for a better outcome versus a worse outcome is identical wherever the scale is dichotomized. Each patient contributes to the underlying analyses and we obtain an overall estimate of the shift in outcome across the GOS called a “shift analysis [19]. Depending on the distribution of other continuous outcomes, an independent samples *t*-test or log-transformed or Mann–Whitney *U* test will be used as appropriate. Binary outcomes will be compared using Fisher’s exact probability test; 95% confidence limits will be calculated and P-values <0.025 for both of the co-primary outcomes will be considered statistically significant, that is, a cumulated family-wise error risk of 0.05 at the most will be allowed if statistical significance is declared.

## Discussion

One of the main challenges in evaluating possible effects of a novel drug on cerebral vasospasm is the choice of outcome measures. Mortality and morbidity are not suitable as primary endpoints of a pilot trial due to the required sample size. Clinical parameters, transcranial Doppler, CT angiography, digital subtraction angiography, invasive monitoring and CT perfusion all have their strengths and weaknesses. The ideal parameter would be continuous, non-invasive, specific and easily quantified, but such a parameter does not exist because of the complex nature of vasospasm. Radiographic vasospasm without clinical symptoms is frequent and clinical symptoms (DIND) can be seen without evidence of arterial narrowing. In this trial we have chosen cerebral perfusion as the primary outcome measure as this seems to be a possible surrogate outcome measure with high biological relevance. However, the secondary outcome measures in this explorative trial are also considered important.

One could argue that in order to attempt to prevent the development of vasospasm, intervention during the entire period at risk (i.e. days 1–21) would be reasonable. However, the logistics at our institution do not allow for all patients to be monitored sufficiently for the entire 3-week period. Furthermore, CT perfusion scans must be performed in the day time (and not on weekends) for logistical reasons, and baseline values must be obtained before the intervention is initiated. Consequently, we have chosen to test the interventions in the period with the maximum risk of vasospasm (days 5–10).

The maximum dose of 2 ng/kg/min in the present trial is relatively low compared to some other clinical indications for the use of prostacyclin such as pulmonary hypertension, where doses of 20 ng/kg/min or more are used. In animal experiments it has been shown, however, that doses in the range of 0.5-2 ng/kg/min have physiological effects by improving microcirculation and reducing microvascular permeability in the brain, skeletal muscle and intestines [12,14,15,20,21]. This has also been shown in the human brain [14,15,20]. A previous study suggesting beneficial effects on vasospasm with prostacyclin used doses in the range of 0.5-1 ng/kg/min [17]. These doses also fulfil the theoretical rationale behind the administration of prostacyclin to patients with SAH, to compensate for the decreased endogenous production. A dose of 1–2 ng/kg/min may therefore be effective and at the same time avoid the well-known adverse effects due to higher doses, such as hypotension, bleeding, abdominal pain and headache, some of which are unacceptable for our trial patients.

## Trial status

Currently including patients

## Competing interests

The authors declare that they have no competing interest.

## Authors' contributions

RR contributed to the conception and design of the trial, and data acquisition, and drafted the manuscript. TS designed the CTP/CTA protocols. JW, BR, PG, JS and NO made contributions to the conception and design of the trial and critically revised the manuscript for important intellectual content. All authors read and approved the final manuscript.
